# Supplementary figures and images for: Achieving clinically optimal balance between accuracy and simplicity of a formula for manual use: Development of a simple formula for estimating liver graft weight with donor anthropometrics
Source: PLoS One. 2023 Jan 20;18(1):e0280569. doi: 10.1371/journal.pone.0280569 (PMC9858735; doi:10.1371/journal.pone.0280569)

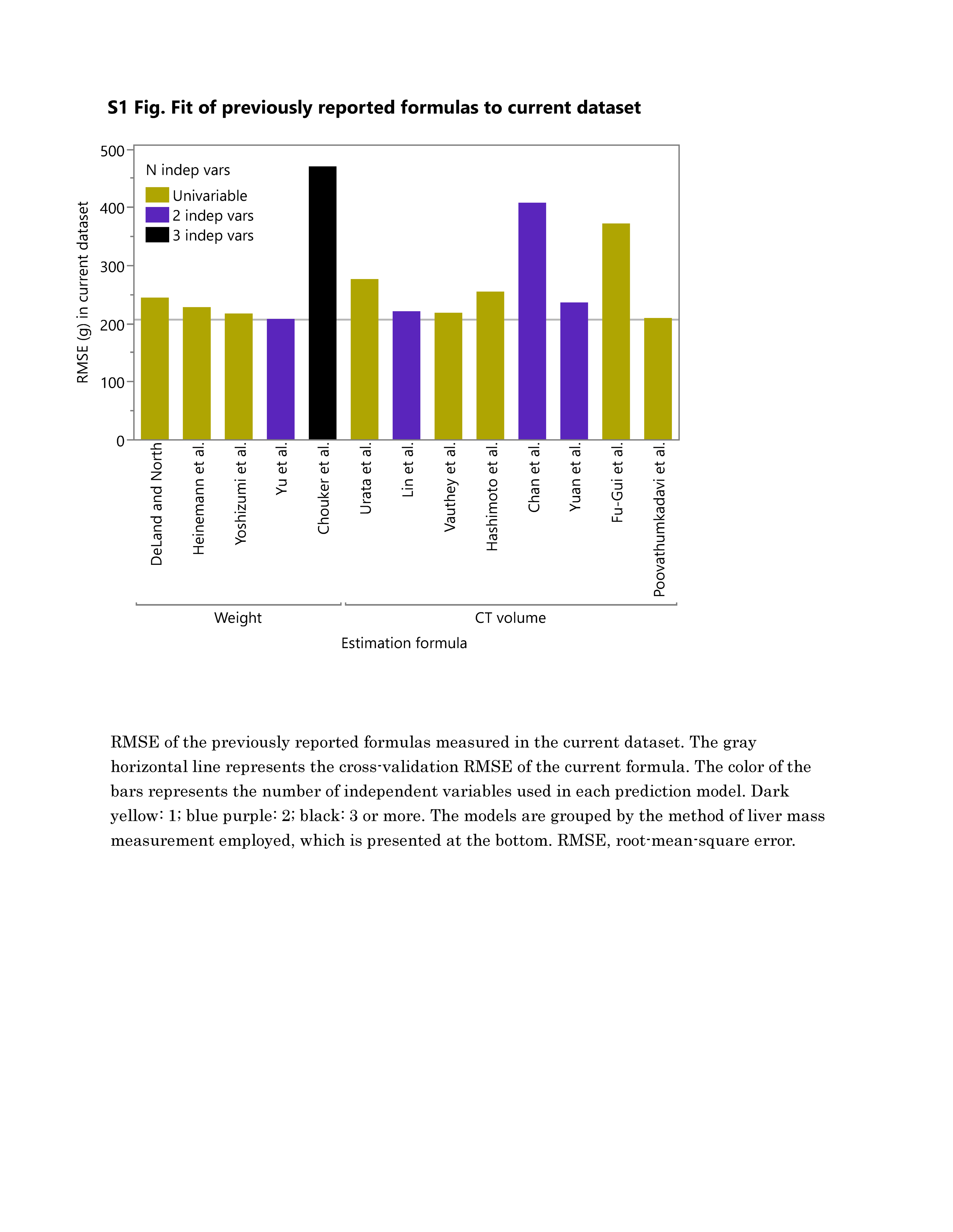

Supplement: S1 Fig — RMSE of the previously reported formulas measured in the current dataset. The gray horizontal line represents the cross-validation RMSE of the current formula. The color of the bars represents the number of independent variables used in each prediction model. Dark yellow: 1; blue purple: 2; black: 3 or more. The models are grouped by the method of liver mass measurement employed, which is presented at the bottom. RMSE, root-mean-square error. (TIF) [file pone.0280569.s001.tif]

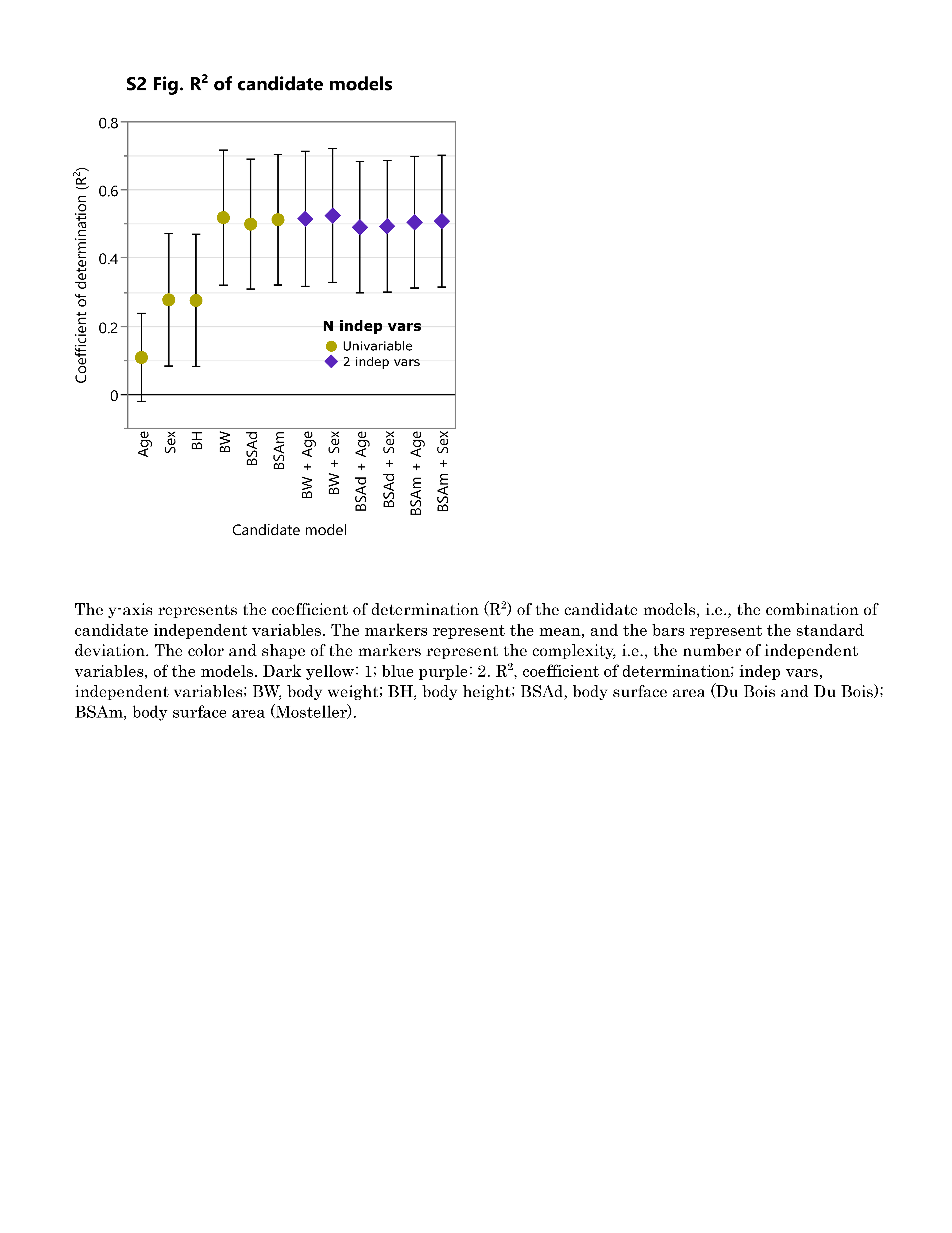

Supplement: S2 Fig — The y-axis represents the coefficient of determination (R2) of the candidate models, i.e., the combination of candidate independent variables. The markers represent the mean, and the bars represent the standard deviation. The color and shape of the markers represent the complexity, i.e., the number of independent variables, of the models. Dark yellow: 1; blue purple: 2. R2, coefficient of determination; indep vars, independent variables; BW, body weight; BH, body height; BSAd, body surface area (Du Bois and Du Bois); BSAm, body surface area (Mosteller). (TIF) [file pone.0280569.s002.tif]
