# Supplementary material for: Achieving clinically optimal balance between accuracy and simplicity of a formula for manual use: Development of a simple formula for estimating liver graft weight with donor anthropometrics
Source: PLoS One. 2023 Jan 20;18(1):e0280569. doi: 10.1371/journal.pone.0280569 (PMC9858735; doi:10.1371/journal.pone.0280569)
Supplement: S1 Text — (DOCX) [file pone.0280569.s003.docx]

# S3 Text. Review of previously reported formulas for estimating liver mass.

## Variation of previously reported estimation formulas regarding their in-sample R^2^ and R^2^ in the current sample

This is the first study to examine the fit of previously reported estimation formulas for liver mass on an independent sample of liver-graft weight measurement using R^2^ and RMSE (Table 1, Figure 5, and Figures S1). The results showed variable accuracy, which was consistent with the findings of Pomposelli et al., who assessed the accuracy of published estimation formulas for WL graft mass using computed tomography (CT)-derived SLV.^(22)^

## Differences between populations as a reason for the poor fit of the previously reported formulas on the current data

No apparent correlation was observed between the reported in-sample R^2^ and R^2^ (or RMSE) in the current WL cohort (Table 1, Figure 5). One potential reason for this discrepancy is population differences. A previous study has shown that an estimation formula derived from “Western” living donors did not conform to Japanese living donors.^5^ The difference of mean WL graft weight or SLV among these reports (Table 1) also suggest biological difference. In this study, no apparent correlation was observed between the overall fit or calibration of the previously reported estimation formulas and their country of data collection (Table 1, Figures 5, 6, and S1).

In addition to “race” (*e.g.*, “Western” *vs*. “Asian”), there are some other known differences between populations on which previous and current estimation formulas have been developed. For example, while the current formula was developed using an adult-only sample, some previous models were developed based on samples that included individuals younger than 18 years. ^(2, 5, 7, 10, 13, 22)^ Specifically, Urata et al. reported that BSA is more suitable than BW for estimating liver volume when the population includes both adults and young individuals (<16 years).^(2)^

Similarly, differences in methods for liver mass measurement (Table 1) might contribute to the observed discrepancy between the reported in-sample R^2^ of previous estimation formulas and their R^2^ in the current sample. When applied to the current sample, there was no apparent correlation between model fit or calibration of the previously reported models and their liver mass measurement method (Table 1, Figures 5, 6, and S1).

## Estimating the external validity of clinical estimation models

Another possible reason for the observed discrepancy between the reported in-sample R^2^ of the previous models and their R^2^ in the current cohort is the failure of in-sample R^2^ to represent their external validity, which is a well-known issue with in-sample measures of model fit. Unlike previous studies, we addressed this issue by examining the external validity of the models by cross-validation. The authors believe that the results of appropriately designed model validation should be routinely reported in clinical studies involving estimation/prediction. Measures of estimated external validity published with each estimation formula would allow examining the feasibility of applying estimation formulas across samples, distinguishing the influence of overfitting from that of population difference.

## Assessing model calibration in independent samples

The actual *vs.* estimated plots (Figure 6) show that the observed variation in estimation accuracy on the current cohort among the previously reported models, represented by their R^2^, largely reflects variations in their calibration, not discrimination. This highlights the importance of assessing the performance of estimation models in independent samples, separating their calibration and discrimination.^23^
