# Supplementary material for: Achieving clinically optimal balance between accuracy and simplicity of a formula for manual use: Development of a simple formula for estimating liver graft weight with donor anthropometrics
Source: PLoS One. 2023 Jan 20;18(1):e0280569. doi: 10.1371/journal.pone.0280569 (PMC9858735; doi:10.1371/journal.pone.0280569)
Supplement: S2 Text — (DOCX) [file pone.0280569.s004.docx]

# S4 Text. Variable selection in liver mass estimation

## BW *vs.* BSA for estimating WL mass

There is no consensus on the best variable for estimating WL graft mass among demographic and anthropometric parameters. BW and BSA, both Du Bois and Du Bois’ and Mosteller’s, have been reported to yield the highest accuracy when used as a single independent variable for estimating WL mass (Table 1). Comparison of accuracy among such candidate models have not been quantitatively presented in the previous reports.

We demonstrated the relative accuracy of these candidate models, minimizing their variation caused by sampling, through “inner” cross-validation (Figures 2, 3, and S2). When averaged through the “inner” CV, among three univariable formulas using BW, Du Bois and Du Bois’ BSA, and Mosteller’s BSA, the BW formula had the lowest CV RMSE. However, the difference in CV RMSE of these three formulas were smaller than 5%, i.e., the difference in accuracy of these three univariable formulas were clinically negligible.

Furthermore, we quantitatively examined variation in model selection by employing “outer” cross-validation. While the univariable BW model was selected as the final model, models using Mosteller’s BSA were selected in 5% of random subsamples, even with “voting” embedded in the model selection procedure, which enhanced the difference in frequency between models. This also indicates that the univariable model with Mosteller’s BSA is not much less accurate than the BW model.

## Age and sex in estimating liver mass

Since Yonemura et al. reported that SLV was significantly overestimated in persons aged <30 years^(16)^, several studies have demonstrated that age has an independent effect on WL volume.^(7, 11, 17)^ In this study, in persons ≥18 years old, adding age as the second independent variable to estimation formulas based on BW or BSA was not observed to improve their accuracy to a clinically meaningful degree (Figures 2 and S2). This finding is aligned with Vauthey et al.’s report that age could be negligible in an estimation formula for WL mass.^(5)^

Two of the thirteen published formulas for estimating liver mass included sex as an independent variable.^(7, 9)^ In this study, combining sex with BW or BSA as independent variables did not lead to clinically higher accuracy for estimating WL weight compared to those using BW or BSA only (Figures 2 and S2).

## Liver mass measurement method

Directly measured graft weight, not CT-derived liver volume, was used in this study as a method of liver mass measurement. While using CT-derived liver volume to fit liver-mass estimation formulas may allow the incorporation of a population similar to the candidate liver donors, one advantage of using direct measurements of liver graft weight is its established value as a predictor of the recipient’s prognosis.^(18, 19)^ Conversely, no prognosis prediction models have been reported, to our knowledge, that incorporate CT-derived SLV. CT-derived SLV is reported to overestimate WL graft volume, with an error ratio of 5-25% when compared with the actual graft weight measured on the back table.^(20, 21)^
